# Plasticity of the Melanocortin System: Determinants and Possible Consequences on Food Intake

**DOI:** 10.3389/fendo.2015.00143

**Published:** 2015-09-14

**Authors:** Danaé Nuzzaci, Amélie Laderrière, Aleth Lemoine, Emmanuelle Nédélec, Luc Pénicaud, Caroline Rigault, Alexandre Benani

**Affiliations:** ^1^Center for Taste and Feeding Behaviour, CNRS (UMR6265), INRA (UMR1324), Université de Bourgogne-Franche Comté, Dijon, France

**Keywords:** synaptic plasticity, melanocortin system, food intake, obesity, AgRP neurons, POMC neurons

## Abstract

The melanocortin system is one of the most important neuronal pathways involved in the regulation of food intake and is probably the best characterized. Agouti-related peptide (AgRP) and proopiomelanocortin (POMC) expressing neurons located in the arcuate nucleus of the hypothalamus are the key elements of this system. These two neuronal populations are sensitive to circulating molecules and receive many excitatory and inhibitory inputs from various brain areas. According to sensory and metabolic information they integrate, these neurons control different aspects of feeding behavior and orchestrate autonomic responses aimed at maintaining energy homeostasis. Interestingly, composition and abundance of pre-synaptic inputs onto arcuate AgRP and POMC neurons vary in the adult hypothalamus in response to changes in the metabolic state, a phenomenon that can be recapitulated by treatment with hormones, such as leptin or ghrelin. As described in other neuroendrocrine systems, glia might be determinant to shift the synaptic configuration of AgRP and POMC neurons. Here, we discuss the physiological outcome of the synaptic plasticity of the melanocortin system, and more particularly its contribution to the control of energy balance. The discovery of this attribute has changed how we view obesity and related disorders, and opens new perspectives for their management.

## Introduction

Brain plasticity refers to the natural capacity of the brain to modify its structure and function through experience. This attribute relies on the property of neurons to adjust their responsiveness, molecular content, and connections as a result of activity. At the level of synapses, plasticity includes functional changes that strengthen or weaken existing synapses – by changing probability of neurotransmitter release and conductance and quantity of post-synaptic receptors – as well as structural changes that involve synapse formation and elimination (1). The hypothalamus, which ensures long-term stability of the inner milieu, is a brain area prone to synaptic plasticity. Neurotransmission in numerous neuronal circuits located in the hypothalamus varies in response to changes within the body and in the environment. This Review focuses on the structural forms of synaptic plasticity in the melanocortin system of the hypothalamus, one of the main neuronal circuits that control appetite and energy homeostasis. We also discuss recent findings suggesting that loss of plasticity in this circuit might compromise its function and confer risk for obesity and related disorders.

## Overview of the Melanocortin System

Feeding behavior is controlled by several homeostatic brain circuits that promote food intake or suppress appetite according to metabolic needs, and by cognitive structures involved in hedonic hunger, eating habits, and emotional processing of the meal context (2). The melanocortin system is one of the most important neuronal pathways involved in the regulation of food intake and is probably the best characterized (3–7). This circuit includes (i) neurons expressing melanocortin receptors (MCR), namely central MC3R and MC4R subtypes, (ii) neurons that express MCR agonists, such as the melanocortin peptide called α-melanocyte-stimulating hormone (MSH), which derives from the proopiomelanocortin (POMC) precursor protein, and (iii) neurons that express MCR antagonists, such as the high-affinity ligand agouti-related peptide (AgRP).

While MC3R and MC4R target neurons are widely distributed through the brain, they are highly enriched in specific brain areas controlling energy balance (8–10). The role of MC3R in energy homeostasis is not well understood yet, but MC3R neurons likely contribute to behavioral adaptation to fasting and nutrient partitioning (11, 12). Conversely, MCR4 is clearly involved in several aspects of energy balance, such as feeding behavior, adaptive thermogenesis, and glucose homeostasis (13–17). The anorectic and weight-lowering functions of MC4R have been evidenced using pharmacological and genetic tools in the late 1990s (18, 19). Moreover, mutations in the MC4R gene represent the most frequent genetic form of obesity and are associated with hyperphagia (20–22).

Interestingly, most of MC4R-expressing sites in the brain receive dual antagonistic innervation of stimulatory POMC and inhibitory AgRP fibers (23–25), suggesting that the melanocortin system is finely tuned to maintain energy homeostasis. POMC- and AgRP-expressing neurons are contained in close proximity in the arcuate nucleus of the hypothalamus, but a small cluster of POMC neurons is also found in the solitary tract nucleus (*nucleus tractus solitarii*, NTS) in the brainstem (26–29). One special feature of the arcuate neurons is their position in a brain parenchyma flooded by blood-borne factors and cerebrospinal factors that are released by permeable microvessels of the median eminence and delivered by tanycytes lining the third ventricle (30–33). The ability of arcuate POMC and AgRP neurons to be regulated by circulating hormones, including leptin, insulin, ghrelin, estrogens, glucocorticoids, glucagon-like peptide 1, and peptide YY, and by nutrients makes the melanocortin system sensitive to changes in body’s energy status (34–36). For instance, the adipose-derived signal leptin increases the activity of POMC neurons and inhibits that of AgRP neurons, which is consistent with the anorexigenic effect of this hormone (29, 37). Intrinsic particularities have been found in the melanocortin system based on the neurochemistry, anatomy, and sensitivity of its neuronal components. Arcuate but not NTS POMC neurons co-express the cocaine amphetamine-related peptide (CART), another anorexigenic molecule (38, 39). Moreover, arcuate POMC neurons are likely segregated into different projectional systems through the rostro-caudal axis of the hypothalamus, sending separated bundles of fibers to anterior brain structures (e.g., bed nucleus of the stria terminalis), lateral regions [e.g., paraventricular nucleus of the hypothalamus (PVN), lateral hypothalamus (LH), amygdala], or caudal regions (e.g., periaqueductal gray) (23, 25, 40). Likewise, distinct subpopulations of arcuate AgRP neurons project to different brain regions (41). AgRP neurons co-express the orexigenic neuropeptide Y (NPY) (42, 43), and the neurotransmitter gamma-aminobutyric acid (GABA) that allows direct inhibition of POMC neurons (29). By contrast, arcuate POMC neurons exhibit diverse GABAergic, glutamatergic, and cholinergic neurotransmitter phenotypes (44–48), but the significance of this heterogeneity remains unclear (49). Nonetheless, divergence in the melanocortin system has been evidenced and the current model comprises dissociated sub-circuits that are dedicated to regulating specific parameters of the energy balance. For instance, MC4R-expressing single-minded homolog 1 (SIM1) neurons located in the PVN control appetite, while others in other parts of the brain control energy expenditure (50). Indeed, sympathetic preganglionic cholinergic MC4R-expressing neurons of the intermediolateral nucleus (IML) of the thoracic spinal cord regulate energy expenditure but not food intake (51).

Until recently, it was thought that AgRP neurons promote food intake via GABA- and NPY-mediated inhibition of local POMC neurons, and via AgRP-mediated competitive antagonism on distant MCR-expressing neurons (3, 52). The use of genetically encoded anatomical, optogenetic, and pharmacogenetic tools allowed for the identification of further modes of action of arcuate AgRP and POMC neurons in the control of food intake. In fact, the MCR signaling is not always mandatory for AgRP neurons to mediate their orexigenic effect. This has been exemplified in two different models. The first is based on selective ablation of AgRP neurons in adult mice. This procedure reduces the GABAergic tone from AgRP neurons within the parabrachial nucleus (PBN) of the hindbrain, a visceral and taste-sensing area, which leads to cessation of feeding and ultimately to death by starvation (53–55). In this specific hypothalamus-to-hindbrain circuit, AgRP innervation of the PBN maintains food intake in mice in a melanocortin-independent manner (54). The second model uses chemical- or light-mediated activation of melanocortin neurons in mice. These experiments show that artificial acute and selective activation of AgRP neurons is sufficient to rapidly induce voracious feeding (56, 57). Because this behavioral response is still found in *Lethal yellow* (*A^y^*) mutant mice, whose ectopic expression of the endogenous MCR antagonist Agouti blocks the melanocortin signaling, this indicates that MCR signaling is not necessary for AgRP neurons to initiate feeding (56). Conversely, photo-activation of POMC reduces food intake but the POMC-mediated anorexigenic effect is completely blocked by the *A^y^* mutation showing that POMC neurons require downstream melanocortin signaling to reduce food intake (56). Further studies have indicated that AgRP neurons control distinct phases of eating via specific molecular signaling pathways and neuronal circuits. NPY and GABA transmitters from AgRP neurons are involved in short-term regulation of food intake, whereas the AgRP peptide itself – as well as arcuate POMC neurons – controls food intake through an action on MC4R over a delayed and prolonged period (56, 58–60). A growing body of evidence suggests that AgRP and POMC neurons not only control homeostatic food intake but also motivate feeding behaviors, including desire to eat and food-seeking behavior (57), preference for flavors (61), visceral malaise anorexia (62), and reward-related behaviors (63). Furthermore, these neurons take into account the nutritional value and accessibility of food together with the internal physiological state to produce an anticipatory modulation of the feeding behavior (64). Finally, current model shows that AgRP neurons engage a constellation of parallel circuits and brain relays to control different aspects of feeding behavior, some of which have a redundant capability to drive feeding behavior (41).

## Evidences of Synaptic Plasticity in the Melanocortin System in Adult Brain

Sensory experiences and changes in the inner milieu deeply modify the function of specialized neuronal circuits in the adult brain (1, 65, 66). This capacity to adapt to ever-changing life conditions relies on coordinated molecular and cellular mechanisms, including changes in synaptic strength and in neuronal connectivity. The concept that the mature hypothalamus can undergo morphological plasticity according to variations of the physiological state and in response to hormonal cues is not a recent idea. In the 1980s, synaptic reorganization within the oxytocinergic system has been evidenced during lactation or in response to changes in water homeostasis (67, 68). In the same time, synaptic remodeling has been found in the hypothalamus in response to sex steroid hormones and during the estrous cycle (69, 70). This hormone-dependent synaptic plasticity affects gonadotropin releasing hormone (GnRH) neurons involved in the neuroendocrine control of reproduction (71). During the two last decades, Tamas Horvath and colleagues have clearly established that the melanocortin system is another hypothalamic system whose connectivity is affected by variation in circulating hormones (72). Pioneer studies were based on depletion–repletion paradigms in living animals to control hormone levels combined with in-depth inspection of synaptic organization on arcuate POMC and NPY neurons (73–75). In the first report, the situation in mutant *ob*/*ob* mice lacking leptin was compared to that of wild-type and leptin-supplemented *ob*/*ob* mice (73). Electron microscopic stereology on fixed brain sections combined to *ex vivo* electrophysiological recordings of living brain slices revealed that leptin induces a shift in the composition of afferent inputs on POMC neurons in adult brain, by increasing excitatory synapses apposed on these cells within only 6 h following intraperitoneal injection. Opposite effects were found on NPY/AgRP neurons. Interestingly, leptin-induced synaptic changes are consistent with the anorectic effect of leptin suggesting that a causal relation might exist. Similar strategies were used to evidence remodeling properties of estradiol and corticosterone, whose effects on the connectivity of the adult melanocortin system are still consistent with their physiological actions (74, 75). Further studies have revealed that injection of ghrelin in wild-type mice also causes synaptic remodeling on POMC and NPY/AgRP neurons (73, 76). All these observations clearly indicate the ability of the adult melanocortin to rewire in response to metabolic cues.

## Connectivity of the Melanocortin System is Nutritionally Regulated

Plasticity of the melanocortin system in adult brain has been initially evidenced in response to hormone replacement in depleted backgrounds found in mutant *ob*/*ob*, ovariectomized, or adrenalectomized mice (73–75). This discovery markedly changed our understanding of how hormones regulate the melanocortin system. A second breakthrough was achieved in the early 2010s when it was shown that synaptic rearrangement on POMC and NPY/AgRP neurons naturally occurs in naïve animals in response to changes in the nutritional state.

During positive energy balance, high level of circulating leptin stimulates arcuate leptin-sensitive POMC neurons, which might activate anorexigenic MC4R-signaling in downstream neurons *via* α-MSH release (29, 77, 78). In parallel, leptin blunts the tonic AgRP activity by reducing excitatory inputs on AgRP neurons through an opioid receptor-dependent pathway (78). It is likely the release of β-endorphin from leptin-stimulated POMC neurons that downregulates the glutamatergic activity on AgRP neurons (78).

Food deprivation induces synaptic remodeling in both AgRP and POMC neurons (77–79). Orexigenic ghrelin, which is elevated after fasting (80), promotes excitatory glutamate release onto AgRP neurons (78). Ca^2+^ and AMPK intracellular signaling in pre-synaptic glutamatergic neurons mediates this effect (78). *N*-methyl-d-aspartate (NMDA) receptors and spinogenesis in AgRP neurons are also required to increase AgRP activity during fasting (79). In addition, the inhibitory tone on POMC neurons increases when blood leptin level falls during fasting (77, 78). Fasting-induced inhibition of POMC neurons might result from ghrelin-activated GABAergic AgRP neurons that innervate POMC neurons (29, 58, 77, 81) but is also caused by apposition of new inhibitory terminals on POMC neurons, which are non-AgRP GABAergic neurons (77).

**Figure 1:**
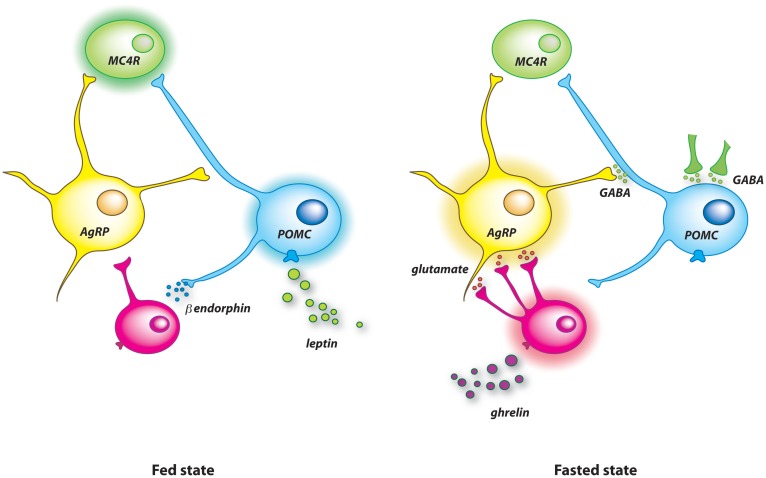
**Model illustrating the synaptic remodeling on arcuate AgRP and POMC neurons after food deprivation**. In the fed state (left panel), high level of circulating leptin stimulates POMC neurons, which probably activate MC4R downstream neurons to limit further food intake. Leptin also causes the release of β-endorphin from POMC neurons. This endogenous opioid blunts the activity of AgRP neurons by reducing the excitatory glutamatergic tone applied on them. Food deprivation raises the level of circulating ghrelin (right panel). This orexigenic hormone promotes apposition of new excitatory synapses on AgRP neurons that increases the activity of these cells. The concomitant drop of blood leptin allows the insertion of inhibitory terminals on POMC cells. These additional inhibitory synapses originate mainly from non-AgRP GABAergic neurons.

Finally, the current model for food deprivation-induced neuronal rewiring implies two opposite signals acting on several cell targets (Figure 1). Ghrelin rewires the melanocortin system during negative energy balance and basal synaptic organization is recovered by the rise of leptin when energy balance is restored. In this model, leptin is viewed as a resetting signal. On the other hand, overfeeding for few days, which is associated with high leptin level, is another nutritional situation that initiates synaptic remodeling in the melanocortin system (82). Precisely, short-term high-fat diet for 3 days increases excitatory inputs on POMC neurons. This observation indicates that synaptic plasticity of AgRP and POMC neurons is not a specific response to fasting and suggests that synaptic remodeling is activated after positive or negative deviation from the metabolic set point. Underlying biological mediators, molecular pathways as well as origin of dynamic synapses probably differ for each situation.

## Astrocytes Regulate the Connectivity of Melanocortin Neurons

Initially viewed as the “brain glue,” i.e., an inert scaffold for neural tissues, and then as non-excitable supporting and nourishing cells for neurons, astrocytes are now considered as key players in neurotransmission (83). Recently, it has been proposed that these glial cells contribute to the brain control of energy balance (84–86). In fact, astrocytes are at the interface between blood vessels and neurons (87). Expression of sensors for metabolic signals, including those for glucose, fatty acids, insulin, leptin, insulin-like growth factor-1 (IGF1), and glucocorticoids, give to astrocytes the ability to integrate changes in the microenvironment that might be communicated to neurons (88–96). Obviously, hypothalamic and hindbrain astrocytes are glucosensitive cells involved in several physiological functions, including glucose homeostasis and gastric motility (97–100). Moreover, leptin regulates the expression of glutamate and glucose transporters in hypothalamic astrocytes (101). Alteration of leptin signaling in astrocytes causes aberrant feeding responses to fasting and to leptin or ghrelin injections (96). Feeding response to high-fat diet is also impaired by disruption of the nuclear factor kappa-light-chain-enhancer of activated B cells (NF-κB) pathway in astrocytes (102). All these data support the idea that astrocytes contribute to the control of food intake. However, the mode of action of astrocytes for modulating activity of feeding circuits is not totally elucidated. Indeed, they act on neurons through a wide variety of mechanisms. These glial cells modulate neuronal excitability and synaptic transmission *via* the clearance of synaptic transmitters and the delivery of signaling compounds, called gliotransmitters, such as protons, lactate, and adenosine (103, 104). For instance, blood osmolality is nutritionally regulated through a ghrelin-dependent astrocytic ATP release on vasopressin neurons (105). Furthermore, it has been shown that astrocytes control food intake by inhibiting AgRP neurons via adenosine A1 receptors (106). Astrocytes also produce bioactive molecules, such as apolipoprotein E (ApoE), the most abundant lipid transporter in the brain, which acts in the hypothalamus as a satiety factor by reducing food intake after central administration (107). Modification of astrocyte coverage at the synapses is another mean to efficiently and rapidly modulate the synaptic strength onto neurons (108–110). Time-lapse imaging shows that perisynaptic astrocytic processes that embrace synapses are highly reactive and motile structures, to an even higher degree than dendritic spines (111). Interestingly, leptin alters the expression of structural proteins, such as glial fibrillary acidic protein (GFAP), actin, and vimentin, in astrocytes, which is accompanied by changes in astrocytes morphology (112). Furthermore, the morphology of astrocytes surrounding arcuate POMC and AgRP soma varies in the adult brain according to nutritional history, metabolic state, and leptin signaling (96, 101, 113). These morphological changes significantly influence the synaptic inputs received by melanocortin neurons, which might affect overall activity of the system and ultimately the feeding behavior (96, 101, 113).

## Physiological Relevance of Synaptic Plasticity in the Melanocortin System

The ability of the brain to change physically and functionally following experience is a way to adapt to changing environment. Thus, rearrangement of pre-synaptic contacts onto AgRP and POMC neurons in response to metabolic cues would serve to adjust the reactiveness of the melanocortin circuit and to finely control feeding behavior according to energy availability. This attribute would avoid stereotyped responses and maladaptive behaviors. For instance, in fasted animals synaptic remodeling in AgRP and POMC neurons might contribute to increase sensitivity for food cues and/or to increase threshold for satiety signals in order to increase meal size following food deprivation. However, such relation has not been established yet. Nevertheless, it is very likely that the plasticity of the melanocortin system is involved in the regulation of food intake and energy homeostasis. First, because the synaptic rearrangement on AgRP and/or POMC neurons naturally occurs in adult animals in response to positive or negative deviation from a metabolic steady state, i.e., situations that require central regulation of energy metabolism and behavior (78, 79, 82). Second, because the synaptic rearrangement on AgRP and/or POMC neurons caused by metabolic imbalance is consistent with the activation of a counter-regulatory response mediated by changes in AgRP and POMC neuronal activity (78, 79, 82). This observation is in line with seminal reports showing that hormones affect AgRP and POMC synaptology in a manner compatible with their effects on food intake and body weight regulation (73–75, 78). Third, because removal of polysialic acid (PSA), a major synaptic plasticity-promoting factor, from the hypothalamus not only inhibits POMC neurons rewiring during overfeeding but also blunts the following normalization of energy intake (82). While the permissive factor PSA was not selectively ablated in POMC cells in this study – but removed in the whole hypothalamus – these results suggest that POMC rewiring induced by dietary fat consumption contributes to the homeostatic control of food intake. Further studies based on cell-specific manipulation of synaptic plasticity in adult animals are definitively needed to characterize physiological consequences of plasticity-related mechanisms caused by changes in the nutritional state.

## Conclusion and Perspectives

Synaptic plasticity of the melanocortin system is currently considered as an adaptive process activated by huge variations in circulating hormones that can be seen during extreme metabolic circumstances, such as 3-day overfeeding and 24-h fasting in rodents. Other plasticity-related processes occur in response to these metabolic challenges. Overfeeding increases cell proliferation in the mouse brain within few days (114). Blocking the early proliferative burst impairs the normalization of energy intake on the short term. In addition, percentage of newly generated cells adopting a POMC-phenotype in the arcuate nucleus is increased on the long term. This suggests that cell renewal is accelerated during metabolic imbalance, and that maturation of newborn cells is nutritionally regulated to maintain energy homeostasis. On the other hand, fasting induces remodeling of the tanycytic barrier and change in permeability of capillaries in the median eminence and the arcuate nucleus of the hypothalamus, a process that modifies blood–brain exchanges and diffusion of blood-borne molecules into the arcuate parenchyma (31). Structural remodeling in brain feeding centers likely strengthens functional plasticity based on pure pharmacological mechanisms within circuits. Indeed, high-fat feeding induces transient suppression of orexigenic neuropeptides and subsequent induction of anorexigenic neuropeptides (115). Secretion of neuropeptides from POMC neurons can be changed selectively, which further increases the plasticity of the melanocortin system. Activation of cannabinoid receptor 1 (CB_1_R) induces β-endorphin release, but not α-MSH, in a subset of POMC neurons, and thereby shifts the function of these appetite-suppressing neurons and increases feeding behavior (116). Interestingly, neurotransmission in the dopaminergic system of the ventral tegmental area is also regulated according to the metabolic state and this regulation involves endocannabinoids too (117). This mechanism might serve to modulate the anticipatory activity and preference for food-related cues. Thus, plasticity of the melanocortin system might be an element of a protecting arsenal based on disseminated structural and functional changes throughout the brain that are engaged during metabolic imbalance.

Metabolic magnetic resonance imaging has shown that the human hypothalamus is competent for plasticity (118). Therefore, it would not be surprising to find in the human brain plasticity-related events similar to those that have been detected in the melanocortin system of laboratory animals. As a corollary, deficiency in the ability to rewire feeding circuits on-demand would confer a risk for maladaptive feeding behaviors, obesity, and related disorders. This hypothesis has been recently strengthened by a meta-analysis of genome-wide association studies for body mass index in human that has brought to the front of the scene the role of the central nervous system in obesity susceptibility, implicating genes related to synaptic function and plasticity (119).

## Conflict of Interest Statement

The authors declare that the research was conducted in the absence of any commercial or financial relationships that could be construed as a potential conflict of interest.
